# Understanding of Lanthanide-Doped Core–Shell Structure at the Nanoscale Level

**DOI:** 10.3390/nano14121063

**Published:** 2024-06-20

**Authors:** Qing Zhao, Xinle Tian, Langtao Ren, Yan Su, Qianqian Su

**Affiliations:** 1 Institute of Nanochemistry and Nanobiology, Shanghai University, Shanghai 200444, China; 2 Genome Institute of Singapore, Agency of Science Technology and Research, Singapore 138672, Singapore

**Keywords:** rare earth, core–shell structures, cation mixing, lattice strain, spectral modulation

## Abstract

The groundbreaking development of lanthanide-doped core–shell nanostructures have successfully achieved precise optical tuning of rare-earth nanocrystals, leading to significant improvements in energy transfer efficiency and facilitating multifunctional integration. Exploring the atomic-level structural, physical, and optical properties of rare-earth core–shell nanocrystals is essential for advancing our understanding of their fundamental principles and driving the development of emerging applications. However, our knowledge of the atomic-level structural details of rare-earth nanocrystal core–shell structures remains limited. This review provides a comprehensive discussion of synthesis strategies, characterization techniques, interfacial ion-mixing phenomena, strain effects, and spectral modulation in core–shell structures of rare-earth-doped nanocrystals. Additionally, we prospectively discuss the challenges encountered in studying the fine structures of rare-earth-doped core–shell nanocrystals, particularly the increasing demand for researchers to integrate interdisciplinary knowledge and utilize high-end precision instruments.

## 1. Introduction

Rare-earth ions are ideal candidates for the upconversion of luminescent materials because of their unique energy level configuration and energy-transferrable real intermediary level [1,2,3,4,5,6]. Researchers are dedicated to enhancing the luminescent properties of lanthanide-doped nanomaterials through various strategies, such as constructing core–shell nanostructures [7,8,9,10,11,12,13,14,15], finely tuning the concentration of active ion doping [16], establishing organic–inorganic hybrid systems [17,18,19,20,21,22,23,24,25,26,27,28,29,30,31], combining metal nanoparticles [32,33,34,35,36,37,38,39,40,41], engineering dielectric nanoparticles [42,43,44], and adjusting host materials [45]. The progress in core–shell nanostructure engineering enables the precise design of rare-earth-doped nanocrystals and the regulation of their optical properties. It also achieves dual control over the morphology and chemical–physical properties of nanomaterials, greatly facilitating the optimization of their optical, electrical, and magnetic properties [46,47,48,49,50]. By skillfully designing the nanoscale core–shell architecture, effective control over the energy transfer pathway can be achieved, resulting in a positive impact on luminescence intensity, spectral band, and lifetime [46,51,52,53].

At the frontier of engineering research and development of rare-earth functional nanomaterials, the pursuit of precise modulation at the atomic level has become a central goal. Controlling the homogeneity and diversity of individual luminescent nanoparticles will lead to breakthroughs in many quantitative measurements and the development of high-performance nano-optical devices and biosensors [54,55]. Future challenges and opportunities focus on the precision synthesis and characterization of heterogeneous core–shell nanostructures for multifunctional integration. In this context, rare-earth-doped heterogeneous core–shell nanostructures have become a key way to unlock this potential. Future research will deepen the construction of heterogeneous structures to achieve ultra-fine modulation of nanomaterial morphology, interfaces, and optical properties for a wider range of applications.

The utility of heterogeneous core–shell structure design as an advanced strategy to modulate the properties of nanomaterials is remarkable. In recent years, scientific researchers have been exploring the phenomenon of cationic interfacial mixing and strain effects induced by lattice mismatch, and this advancement has enabled us to tackle the complex challenges at the atomic level in a more refined manner [56,57,58,59,60,61,62,63,64,65]. This review focuses on the fundamental research of rare-earth core–shell nanomaterials, which includes synthesis and characterization strategies, recent advances in interfacial ion-mixing phenomena and interfacial strain effects, as well as how to utilize the core–shell structure to achieve the efficient modulation of luminescent properties of upconverted nanoparticles.

## 2. Synthesis Techniques and Structural Characterization

There are several methods for synthesizing lanthanide-doped rare-earth nanocrystals, with some of the most common methods including the aqueous thermal method, co-precipitation method, and thermal decomposition method [66,67,68,69,70]. Among these, the aqueous thermal method and co-precipitation method are frequently employed for synthesizing rare-earth core nanocrystals.

### 2.1. Synthesis Techniques

#### 2.1.1. Thermal Synthesis in Water (Solvent)

The hydrothermal and solvent-thermal methods utilize either water or a non-aqueous solvent as the reaction medium in a sealed container like a Teflon-lined hydrothermal autoclave. The container is heated to elevate the temperature and pressure, causing normally insoluble substances to dissolve and crystallize under these reaction conditions. The advantage of this method is that a crystalline powder of higher purity can be obtained at a lower temperature, which is favorable for the synthesis of substances that are heat-sensitive or have poor stability at room temperature. In contrast to other commonly used methods, the aqueous (solvent) thermal method has the disadvantage of conducting the reaction in a closed system, making it difficult to observe morphological changes during the material synthesis.

**Figure 1:**
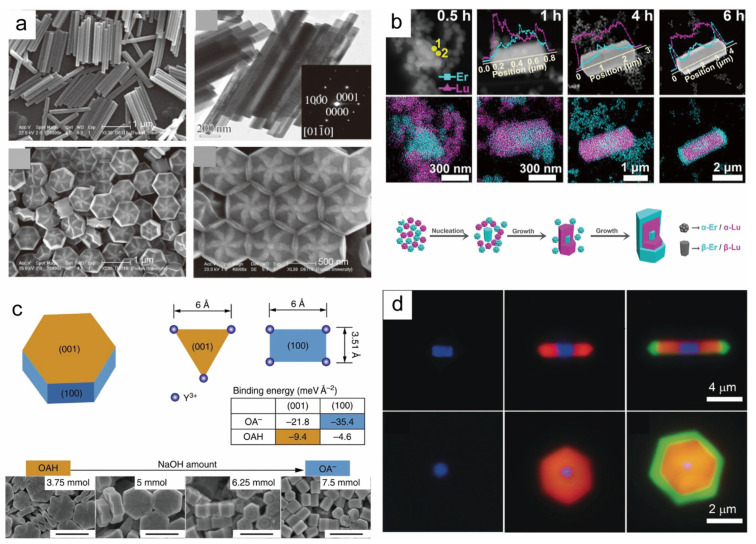
Rare-earth nanocrystals with different morphologies synthesized by the hydrothermal method. (**a**) SEM images of arrays of flower-patterned hexagonal and hexagonal nanorods of β-NaYF_4_. Reproduced with permission from [67]. 2007, WILEY-VCH. (**b**) Schematic diagrams of HAADF-STEM and EDS images and corresponding phase transition processes for different time periods during the synthesis of β-NaREF_4_. Reproduced with permission from [68]. Copyright 2023, American Chemical Society. (**c**) The corresponding schematic diagrams of the directed epitaxial growth mechanism of β-NaYF_4_ nanocrystals, the arrangement pattern and binding energy properties of Y^3+^ ions of OAH (oleic acid hydroxyl) and OA (oleic acid) on the two most stable crystal planes, (001) and (100), as well as the SEM characterization of the synthesis of submicrometer-sized nanocrystals by the hydrothermal method. Reproduced with permission from [71]. Copyright 2016, Nature Publishing Group, scale bar, 500 nm. (**d**) Optical micrographs of rod and plate crystals at different stages under 980 nm laser excitation. Reproduced with permission from [70]. 2016, WILEY-VCH.

The solvothermal method has emerged as the preferred technique for producing core nanocrystals because of its capacity to precisely control the synthesis of rare-earth nanocrystals with various sizes, morphologies, and outstanding optical properties. The synthesis of core nanocrystals establishes a solid groundwork for further developing high-performance core–shell nanoparticles. In the study of solvothermal methods, the liquid–solid solution (LSS) phase transfer and separation technique proposed by Li’s group in 2005 has become a mainstream strategy for synthesizing rare-earth-doped nanocrystals of a wide range of morphologies and sizes [66]. The LSS method is widely used to synthesize various types of rare-earth-doped nanocrystals. Subsequently, Zhao et al. successfully prepared rare-earth-doped NaYF_4_ nanorods, hexagonal nanotubes, and nanodiscs with floral patterns with excellent morphology by using oleic acid (OA) as the ligand and optimizing and improving the LSS method (Figure 1a) [67]. In 2016, Sun et al. took advantage of the fact that hydrothermal methods facilitate termination to control the reaction process, and they used hydrothermal synthesis to observe a series of phase transitions from NaREF_4_ α to β and the nucleation of nanocrystals in the β phase, which revealed a high correlation between the process and the ionic radii of lanthanide elements (Figure 1b) [68]. The same year, Jin et al. controlled the direction of NaYF_4_ epitaxial growth by adjusting the ratio of molecules (OAH) and oleate anions (OA^−^) in the hydrothermal synthesis method (Figure 1c) [71]. In 2017, Chen et al. synthesized monodispersed, particle-size-tunable ZnGa_2_O_4_:Cr^3+^ NIR long afterglow nanocrystals by an improved hydrothermal method. This method solved the technical difficulties in the synthesis of long afterglow nanocrystals [72]. In 2023, Liu et al. reported the synthesis of rare-earth core–shell nanocrystals based on an improved hydrothermal method, which can precisely control the shell thickness and growth direction by controlling the reaction conditions, and they synthesized rare-earth core–shell nanocrystals with a shell thickness of more than 300 nm at a specific crystalline surface, which is a size that is just right for the encoding and decoding process in conventional optical microscopy [70] (Figure 1d).

#### 2.1.2. Co-Precipitation Method

The co-precipitation method is considered as the most commonly used synthesis method due to its advantages such as simple operation, affordability, and no need for advanced instrumentation. The co-precipitation method is a process in which a precipitant is added to a prepared solution of multiple cations and a new material of uniform composition is obtained by a precipitation reaction. This method was first reported in 2002 with the synthesis of downconversion nanocrystals co-doped with lanthanide ions (Eu, Nd, Er, and Ho) [65]. In a typical co-precipitation procedure, the generation of the core and the layer-by-layer encapsulation of the shell layer are often divided into separate operational steps to be accomplished sequentially. In 2007, Yan’s team pioneered the separation of the core and shell growth steps, and this strategy was subsequently further optimized and refined in 2008 by Qian et al. [73,74]. In 2014, Wang et al. simplified the synthesis steps of the co-precipitation method, removed the strict control of the shell precursor dosing rate, and allowed the shell layer thickness to be regulated by adjusting the precursor ratio, which resulted in a simpler and controllable preparation of multilayered core–shell NaGdF_4_ structures. The steps for the preparation of core nanocrystals and core–shell nanocrystals are similar and are mainly divided into three steps: generation of complexes, co-precipitation process, and crystallization into nuclei [75]. Although this strategy may be time-consuming, the co-precipitation method also performs well in the construction of nanomaterials with multilayer shell structures. It is worth mentioning that by precisely regulating the solvent components during the capping process, we can realize the fine control of the epitaxial growth dynamics process of the shell layer, thus achieving the precise regulation of the shell structure. To obtain efficient upconversion luminescence, a relatively high temperature is required to increase the crystallinity of nanoparticles. The co-precipitation method is not only suitable for precise control of stoichiometry to synthesize nanoparticles with various core–shell structures but also for the synthesis of ultrasmall lanthanide-doped nanoparticles with narrow size distributions. In addition, the method shows great potential for combining with other synthesis methods, providing diverse pathways for the synthesis of rare-earth core–shell nanoparticles.

#### 2.1.3. Thermal Injection Method

Thermal injection represents a widely employed technique for fabricating rare-earth core–shell nanocrystals, offering greater efficiency compared to co-precipitation and solvothermal methods. By careful adjusting the quantity of injected shell layer precursor solution, the thickness of the shell layer can be precisely regulated, enabling the preparation of upconversion nanoparticles (UCNPs) with varying shell layer thicknesses. This method can greatly simplify the synthesis of UCNPs by effectively eliminating the steps of cooling, centrifugation, and washing in the traditional step-by-step encapsulation method, which are not required in the traditional method process. In a typical step, the shell layer precursor solution is injected into the reaction solution of the nucleus at high temperature to realize the construction of the core–shell structure in one step.

**Figure 2:**
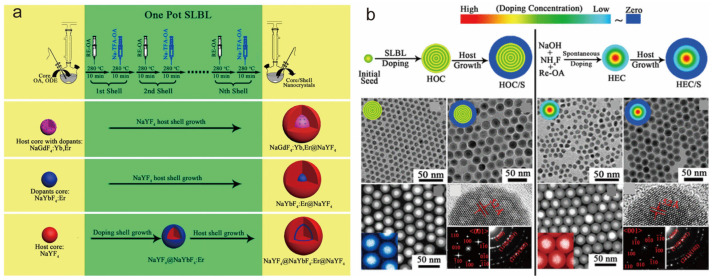
Synthesis of nanoparticles by thermal injection. (**a**) Synthesis procedure and schematic of different types of upconversion nanoparticles (UCNPs) with controllable shell thickness and doping structure prepared using one-pot successive layer-by-layer (SLBL) methodology. Reproduced with permission from [69]. Copyright 2013, American Chemical Society. (**b**) Synthesis steps for the preparation of HOC and HOC/S-type upconversion nanoparticles (UCNPs) using SLBL doping strategy and spontaneous growth process of UCNPs based on one-pot heating method. TEM, HAADF-STEM, HRTEM images, and corresponding FFT and SAED patterns of prepared nanoparticles are shown in the bottom panel. The color bar at the top represents the relative concentration of the dopant elements. Reproduced with permission from [77]. Copyright 2014, American Chemical Society.

Jin et al. used a thermal injection method to encapsulate a NaYF_4_ shell layer on a NaYF_4_ nucleus, and by controlling the reaction parameters, such as the ratio of OA^−^ and OAH and the amount of precursor, the shell layer grew longitudinally on the nucleus and ultimately grew into a nonhomogeneous NaYF_4_ rod [76]. Zhang et al. successfully prepared rare-earth core–shell nanocrystals by using a one-pot successive layer-by-layer (SLBL) strategy using Re-OA (at 140 °C) and NaTFA-OA (at room temperature) as the shell layer precursor solution (Figure 2a). By the successive introduction of shell precursor solutions, this process deposits uniform multishell layers on hexagonal (β) and cubic (α) phase cores. The method can be used for the synthesis of nanocrystals in different phases by simply adjusting the reaction conditions and the type of precursor solution and effectively controlling the shell layer thickness and the dopant ion’s position [69]. In 2014, Zhao and co-workers explored a one-pot sequential layer-by-layer (SLBL) approach as a uniform doping method to produce uniformly doped NaGdF_4_: Yb, Er@NaYF_4_ core–shell nanocrystals, achieving a quantum yield of 0.89 ± 0.05%. This significantly enhanced the luminescence efficiency of UCNPs, as depicted in Figure 2b [77]. Table 1 summarizes the advantages, disadvantages, and key influencing factors of the above synthesis methods.

### 2.2. Structural Characterization

The development of rare-earth core–shell nanostructures has brought new functional properties to rare-earth-doped luminescent nanocrystals compared to homogeneous core nanocrystals, including control over the morphology of the nanomaterials as well as the modulation of their chemical and physical properties. To ensure that the functional properties of these innovative core–shell structures are effectively realized, it is particularly important to validate the characterization techniques for structure synthesis. Moreover, researchers often use a combination of several characterization tools to obtain more accurate conclusions.

Transmission electron microscopy (TEM) characterization is one of the most common means of characterization by which the size and morphology of the synthesized nanoparticles can be observed, and it has a resolution in the order of nanometers. In fact, it can also be used to characterize the synthesis of core–shell structures. Zhang et al. clearly showed the core–multishell nanostructural features of the prepared nanoparticles by TEM images, corresponding to a pure core structure, encapsulating a shell layer, encapsulating up to four layers of shells, presenting uniform sizes and morphologies with average diameters of about 19 nm, 30 nm, 45 nm, 70 nm, and 85 nm. The formation of the nucleus–multishell structure was also demonstrated in conjunction with high-angle annular dark-field scanning transmission electron microscopy (HAADF-STEM) [78].

**Figure 3:**
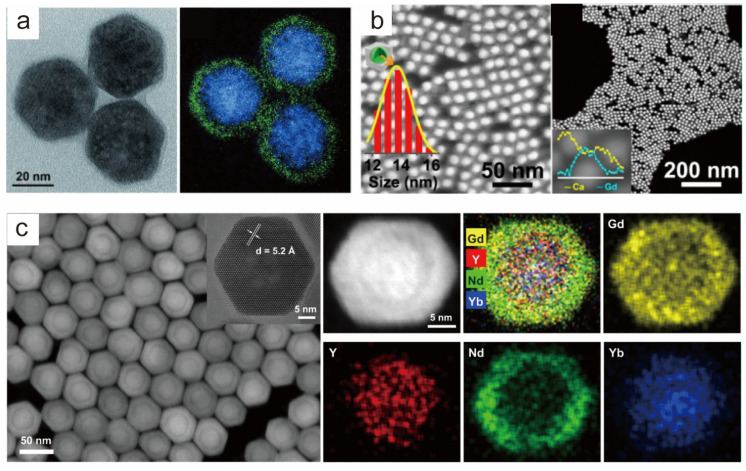
Characterization of core–shell nanocrystals. (**a**) TEM and EELS characterization of the NaYF_4_: Ce@NaYF_4_: Tb structure, confirming the core–shell structure of the nanoparticles. Reproduced with permission from [79]. Copyright 2015 WILEY-VCH. (**b**) HAADF-STEM images and EDS line scanning of β-NaGdF_4_: Yb, Er@CaF_2_ nanoparticles. Reproduced with permission from [80]. Copyright 2017, American Chemical Society. (**c**) HAADF-STEM and EDS-mapping images of a single Gd-CSyS_2_S_3_ nanoparticle, showing the spatial distribution of the Gd, Y, Nd, and Yb elements in the core–multishell structure. Reproduced with permission from [81]. Copyright 2021, Nature Publishing Group.

STEM, as an evolutionary branch of TEM technology, has the ability to characterize and analyze the fine chemical composition and microstructure of materials at the nanometer and atomic levels. When STEM is coupled with an electron loss spectrometer and an X-ray spectroscopy detector, electron energy loss spectroscopy (EELS) and energy dispersive X-ray spectroscopy (EDS) data can be acquired simultaneously, thus revealing the chemical composition and microstructural properties of materials. Electron energy loss spectroscopy (EELS) is a technique for measuring the energy change in incident energetic electrons after inelastic scattering with a sample. EELS has been validated as an effective means to investigate the structural features of lanthanide-doped upconversion nanoparticles (UCNPs) and to reveal the intrinsic connection between their structure and properties. Wang et al. characterized the actual distribution of two elements, Ce^3+^ and Tb^3+^, within the nanoparticles by EELS, which demonstrated that the nanoparticles possessed the designed core–shell structural features (Figure 3a) [79]. Scanning transmission electron microscopy coupled with EDS (STEM-EDS) is a widely used technique for the precise determination of the distribution and migration of ions inside individual nanocrystals (NCs). Yan et al. used a combination of HAADF-STEM and EDS characterization tools to confirm the successful synthesis of a heterogeneous core–shell structure of β-NaGdF_4_@CaF_2_ synthesized by the cation exchange method (Figure 3b) [80]. The successful synthesis of nuclear multishell structures was demonstrated by HAADF-STEM and EDS-mapping analyses in previous studies by our group (Figure 3c) [81].

Time-resolved and steady-state luminescence curves can assist in verifying the synthesis of the core–shell structure. The use of optically inert shells to encapsulate the active nuclei can suppress the excitation energy burst phenomenon caused by external factors, such as ligands, surface defects, and solvent molecules, and significantly enhance the luminescence properties of rare-earth ions. Wang et al. skillfully utilized the unique time-resolved and steady-state luminescence properties of Ce^3+^ and Tb^3+^ to explore the NaYF₄: Ce@NaYF₄: Tb core–shell nanostructures. The lifetime of Ce^3+^ in the nanoparticles was found to be only slightly decreased compared to the Tb^3+^-free shell layer control sample. During the steady-state emission spectroscopy analysis, the emission peak at the ^5^D^3^ energy level was not observed, while the ^5^D^4^ energy level emission feature of Tb^3+^ was presented alone, a phenomenon that indicates that Tb^3+^ ions are not leaking from the shell layer. It is concluded that the interaction between Ce^3+^ and Tb^3+^ is mainly in the region of the core–shell interface. The respective isolated distribution of Ce^3+^ and Tb^3+^ in the core–shell structure is confirmed, i.e., Ce^3+^ is located in the core layer while Tb^3+^ is confined to the shell layer [79].

## 3. Interfacial Ion Mixing

In 2009, Veggel et al. were the first researchers to challenge the previously widely held perception in the scientific community that rare-earth core–shell nanocrystals have a well-defined interface. In 2015, Haase et al. provided spectroscopic evidence for Veggel’s conjecture, finding that a large amount of Eu^3+^ in the nucleus diffuses into the shell layer during shell formation, a groundbreaking discovery that challenged the then widely held scientific perception that rare-earth core–shell nanocrystals have a well-defined interface. Shell nanocrystals with a clear interfacial separation were challenged by this groundbreaking finding [82]. In recent years, the emergence of relevant studies by a number of researchers has further confirmed the prevalence of cation mixing at the interfaces of core–shell structures. The accumulation of this evidence has completely overturned the traditional cognition held by academics for a long time in the past that core–shell structures should have clear interfaces, marking a brand-new stage in the understanding of the interfacial properties of core–shell nanomaterials.

**Figure 4:**
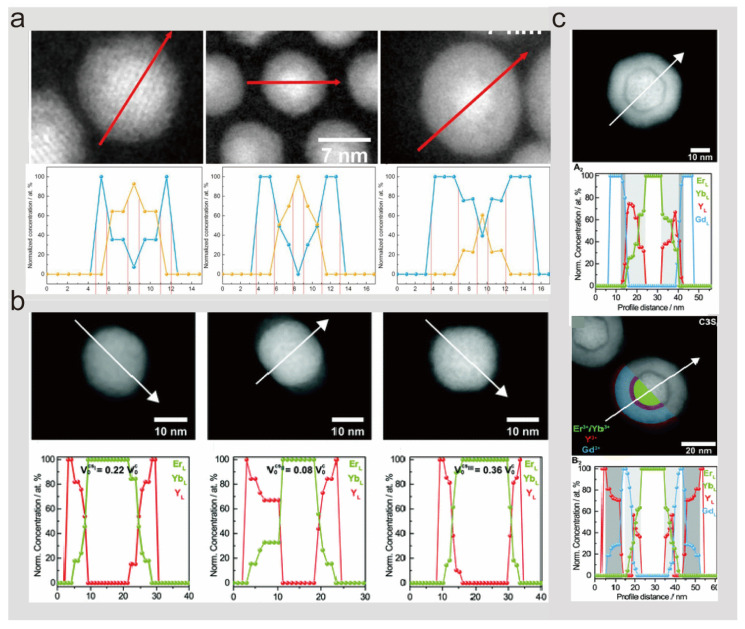
(**a**) HAADF-STEM and energy-dispersive X-ray line scanning spectra of NaGdF_4_: Yb: Er@NaYF_4_ with different thicknesses of epitaxial shell layers, indicating ionic interface mixing. Reproduced with permission from [58]. Copyright 2017, American Chemical Society. (**b**) HAADF-STEM and energy-dispersive X-ray spectroscopy of NaEr_0.8_Yb_0.2_F_4_/NaYF_4_ core–shell structures synthesized by three different shell deposition methods, indicating the presence of interfacial ion mixing. (**c**) HAADF and energy-dispersive X-ray spectroscopic characterization of NaEr_0.8_Yb_0.2_F_4_@NaYF_4_@NaGdF_4_ and NaEr_0.8_Yb_0.2_F_4_@NaYF_4_@NaYF_4_ nanostructures, demonstrating interfacial ion mixing. Reproduced with permission from [60]. 2019, Royal Society of Chemistry.

In exploring this phenomenon, the work of the research team led by Damien Hudry over the past few years has been exemplary. They have continued to delve deeper into this field with a series of studies that are both comprehensive and insightful. In 2017, Damien Hudry et al. investigated core–shell nanocrystals of smaller sizes (~10 nm) by employing inert NaYF_4_ shell layers of varying thicknesses to encapsulate the active nuclei. As obtained from the HAADF-STEM characterization results with different thicknesses of shell layers, the nuclei are not always in the geometric center of the core–shell nanocrystals, but a slight shift is observed. And by the results of the EDS line sweep of the nanocrystals, the concentration profiles and schematic diagrams obtained from the normalization based on the characterization results clearly show that there is no clear and distinct interface between the outer shell and the inner core for all the core–shell structures (Figure 4a). And it is obtained that the content of NaYF_4_ in the mixed interfacial region increases as the thickness of the shell layer increases [58]. In 2018, Damien Hudry and collaborators carried out a further study on the mixing of ions at the interfaces of large-sized nanocrystals with a different number of layers (20–50 nm). Firstly, three different methods were used for the shell coating of pre-prepared NaEr_0.8_Yb_0.2_F_4_, the three core–shell structures synthesized had different compositions and morphologies, and more importantly, interfacial ion mixing existed in all the three core–shell structures as derived from the EDS line-scan results (Figure 4b). The core–shell structures synthesized by method I were then continued to be clad with one and two shell layers. Surprisingly, energy-scattering X-ray spectroscopy analysis revealed that the nucleus is not strictly confined to the geometric center when the second shell layer, the NaGdF_4_ shell layer, is encapsulated; instead, interfacial ion mixing between the nucleus and shell is observed. Similarly, interfacial ion mixing likewise occurs in the third shell layer NaYF_4_ cladding (Figure 4c) [60]. In 2021, it was further revealed that changing the synthesis method of the shells can control the mode of ion mixing at the core–shell interface and also adjust the range of the mixed interface according to the experimental conditions. In addition, the use of different types of interfacial modes can change the photoluminescence properties of heterogeneous core–shell nanocrystals [59]. This series of studies not only deepens the understanding of ionic mixing phenomena in core–shell structures but also opens up new avenues for the precise control of the atomic-scale organization of heterogeneous core–shell structures and thus optimizes their properties, marking a major breakthrough in the field of core–shell lanthanide heterogeneous core–shell research.

## 4. Interfacial Stress

Core–shell nanocrystals offer the opportunity to create materials with customizable physicochemical properties due to their diversity of components and structural tunability. The process of heterogeneous epitaxial shell layer growth involves the simultaneous deposition of shell layer material along all crystallographic orientations of the nanocrystal surface. This results in variations in interfacial strain energies accumulated on different crystallographic surfaces, ultimately causing the anisotropic growth phenomenon of the shell layer. Lanthanide-doped heterogeneous core–shell nanocrystals have lattice mismatches due to the difference in lattice parameters of the core and shell layer ions, resulting in mismatch strain.

In 2013, Lee et al. uncovered that the anisotropic growth of rare-earth core–shell nanostructures might stem from factors like ligand adsorption on the crystal surface and core–shell lattice mismatch. This is evidenced by the observation that the lateral growth rate of nanocrystals synthesized in the oleic acid system exceeds the axial growth rate, and the presence of lattice mismatch leads to the formation of an elliptical shape of the shell layer. Such findings are crucial for elucidating the luminescence mechanism and furthering research on the luminescence mechanism of rare-earth-doped nanocrystals [64]. This work highlights the indirect control of the degree of lattice mismatch by modulating the synthesis environment and optimizing the choice of core–shell materials as a means to effectively regulate the internal strain and final morphology of nanocrystals. Through these strategies, desired lattice mismatches can be purposefully induced to design the growth paths and final morphology of nanomaterials at the molecular level, which is crucial for the development of rare-earth-doped nanomaterials with specific optical properties.

Then, in 2014, van Veggel et al. advanced the understanding further by conducting multiple sets of experiments. They demonstrated that when the core and shell lattice mismatches are comparable, they result in positive mismatches (shell stretching) and negative mismatches (shell compression). This leads to variations in the morphology and structure of the core–shell structure, with the tensile shell layer aligning with the core shape and the compressive shell layer exhibiting anisotropic growth [62]. This study reveals the use of lattice mismatch asymmetry as a tool for “shape engineering”, where the stretched shell layer tends to maintain morphological coherence with the core while the compressed shell layer contributes to anisotropic growth, suggesting that the targeted design of core–shell nanoparticle growth modes can be achieved by precisely controlling the lattice mismatch. This strategy provides a new way for the synthesis of nanomaterials, i.e., the precise control of macroscopic morphology and properties through the tuning of lattice parameters at the microscopic scale, and lays the foundation for the development of nanomaterials with special optical, electrical, or other physicochemical properties. Thus, their study not only deepens the understanding of strain effects on core–shell structures, but also provides important strategic guidance for subsequent material design and synthesis.

**Figure 5:**
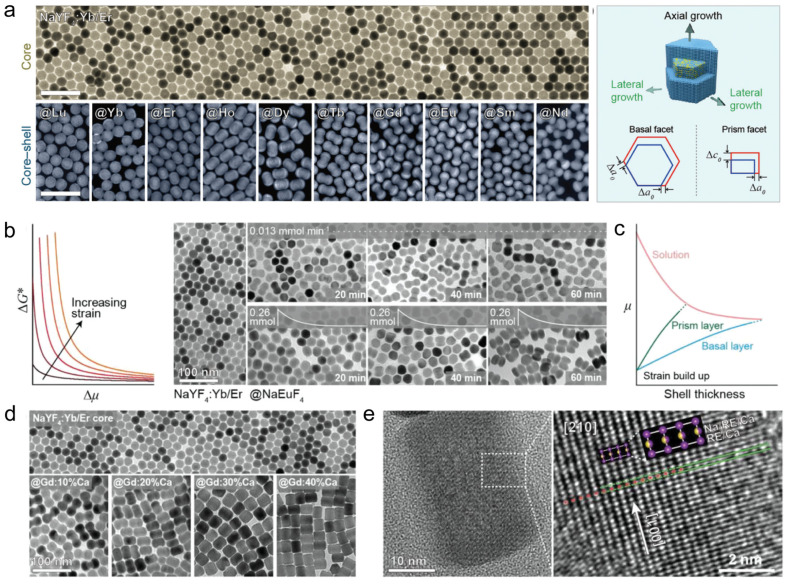
Interfacial stress. (**a**) TEM image of NaYF_4_: Yb/Er core nanoparticles, and HAADF-STEM images of NaYF_4_: Yb/Er@NaREF_4_ nanoparticles with schematic diagrams of lateral and axial growth. Scale bars are 100 nm. (**b**) The relationship between the activation energy-dependent chemical potential and strain during strain growth and TEM images of NaYF_4_: Yb/Er@NaEuF_4_ when changing the precursor injection conditions. (**c**) The thickness versus chemical potential and strain during shell growth. (**d**) TEM images corresponding to the core–shell structure of NaYF_4_: Yb/Er@NaGdF_4_:X%Ca doped with different Ca^2+^ concentrations. (**e**) HRTEM image of NaYF_4_: Yb/Er@NaGdF_4_:X%Ca (40%) taken along the 210-axis, selected as the bending deformation of the (001) crystal surface at the core–shell interface. Reproduced with permission from [61]. 2019, WILEY-VCH.

Wang’s group conducted an in-depth investigation of the effect of anisotropic interfacial strain on the growth behavior and optical properties of sodium rare-earth fluoride core–shell nanoparticles. They used the hexagonal phase NaYF_4_: Yb/Er as the core to encapsulate a series of NaREF_4_ (RE=Nd, Sm, Eu, Gd, Tb, Dy, Ho, Er, Yb, Lu, and Y) shell layers and revealed the evolutionary law of mismatch strain on the morphology of core–shell nanoparticles (Figure 5a). The mismatch strain in NaYF_4_: Yb/Er@NaEuF_4_ core–shell nanocrystals was determined by HRTEM, Fourier transform, and other characterization means. To further precisely regulate the anisotropic growth, accurate control of the anisotropic growth was achieved by adjusting the precursor concentration, precursor injection rate, and the introduction of Ca^2+^ doping in the shell layer (Figure 5b–e). They also found that the enhanced strain energy induced by the lattice mismatch caused the nanoparticles to exhibit a sharper optical responsiveness to changes in ambient temperature [61]. Since strain effects are prevalent in heterogeneous epitaxial core–shell nanocrystals, the results of this study provide a theoretical basis for the design of subsequent experiments. They adopted three key technological measures to achieve precise control of the anisotropic growth: (1) dynamic tuning of precursor concentration; (2) optimization of precursor injection rate; and (3) Ca^2+^ doping in the shell layer. These strategies not only realized the active regulation of strain effects during epitaxial growth but also accidentally discovered the lattice mismatch-induced strain energy enhancement, which enabled the nanoparticles to exhibit a sharper optical response to small changes in ambient temperature, a finding that further expands the potential of strain regulation for applications in areas such as environmental sensing.

Subsequently, Wang et al. proposed an epitaxial growth strategy to modulate the anisotropic interface of large-size core nanocrystals. They employed the characteristic of lattice bending and tilting in rod-shaped nanoparticles with significant aspect ratios to facilitate strain relaxation and epitaxial growth processes efficiently. A NaGdF_4_: Ca shell layer with a large lattice mismatch was successfully grown on NaYbF_4_: Tm nanorods at 300 °C, and a NaNdF_4_: Yb shell layer with a high surface coverage was successfully capped with NaGdF_4_: Ca as a transition layer. The results provide an important strategy and practical guidance to address the challenge of the low coverage of epitaxial shell layers in hexagonal crystalline phase core–shell nanocrystals due to strain problems, and they mark an important step in the field of the precision engineering of nanomaterials [63].

In 2022, Sun’s team developed heterostructures combining rare-earth upconversion luminescent nanoparticles with EuSe semiconductors. They innovatively employed a cation-exchange strategy to address the lattice mismatch issues typically faced in conventional epitaxial growth. Large lattice mismatches typically hinder high-quality epitaxial growth, yet Sun’s team effectively addressed this issue by devising a buffer layer formation mechanism. This approach involved the strategic exchange of Eu^3+^ ions with other rare-earth cations, easing the lattice parameter transition and reducing strain energy at the core–shell interface. Consequently, this facilitated the seamless epitaxial growth of EuSe semiconductors on rare-earth upconverted nanoparticle surfaces. The technique leverages a chemical exchange process as a gentle and precise method to dynamically adjust lattice matching at the interface, thereby naturally regulating strain distribution and preventing severe mismatches and potential structural defects from direct epitaxy. This method of strain modulation not only ensures high-quality heterostructure synthesis but also preserves the stability and optical properties of the materials by alleviating stress caused by lattice mismatches [83].

These studies demonstrate that mismatch strain in core–shell nanostructures has been effectively addressed and exploited through diverse strategies such as the optimization of synthesis conditions, precise regulation of mismatch degree, fine management of growth kinetics, and innovative chemical exchange techniques, providing a rich theoretical and practical basis for the design of nanomaterials with specific properties. These strategies not only advance a deeper understanding of the mismatch strain effect but also open up new avenues for the development of novel nanomaterials for optoelectronics, sensing, and other high-tech applications.

## 5. Luminescence Modulation

### 5.1. Upconversion Luminescence Mechanism

#### 5.1.1. Excited State Absorption (ESA)

In 1959, Bloembergen et al. first introduced the concept of the excited state absorption (ESA) phenomenon, which involves rare-earth ions jumping from the ground-state energy level to the excited-state energy level after undergoing successive two- or multiphoton absorptions. Subsequently, these ions release the accumulated energy back to the ground state via optical radiation [84]. In order to ensure the high efficiency of the ESA process, the ions need to satisfy a series of stringent conditions, i.e., they possess a large absorption cross section, energy level stabilization of the absorbing electrons, and a long intermediate excited state lifetime. However, it is worth noting that a high doping concentration may induce significant nonradiative cross-relaxation, thus weakening the emission intensity. Therefore, the ESA process is more likely to be successful at lower doping levels. These stringent requirements limit the practical application potential of ESA processes in a wider range of fields.

#### 5.1.2. Energy Transfer Upconversion (ETU)

In 1966, Auzel introduced the energy transfer upconversion (ETU) process, which proved to be an efficient mechanism for achieving upconversion luminescence in rare-earth-doped nanomaterials [1]. Compared to ESA, the ETU mechanism is significantly different and centers on its reliance on the synergistic action of two ions: a sensitizer ion with a high absorption cross-section and an activator ion with matched energy levels. Upon excitation by low-energy photons, sensitizer ions can capture the excitation energy and jump to their matched excited states. Immediately thereafter, the sensitizer efficiently transfers the absorbed energy to the activator through a non-radiative energy transfer mechanism.

#### 5.1.3. Co-Operative Sensitization Upconversion (CSU)

The synergistic upconversion mechanism describes the ability of luminescent ions to directly reach the high energy state in the absence of the highest excited state or intermediate substable state energy levels and subsequently emit shorter wavelength photons. Despite some similarities with ETU, the upconversion efficiency of co-operative sensitization upconversion (CSU) is significantly lower, evidenced as being four to five orders of magnitude lower. In a typical CSU process, the excitation energy of two neighboring Yb^3+^ ions is transferred simultaneously to either Tb^3+^ or Eu^3+^ ions. Compared to the luminescence from the high energy level of Er^3+^ or Tm^3+^ during the ETU process, the luminescence of Tb^3+^ or Eu^3+^ during the CSU process is less affected by the harmful surface effect. This is due to the large energy gap between the emission energy level of Tb^3+^ or Eu^3+^ and its next lower energy level, making them show unique advantages and promising prospects for bio-detection and bio-imaging applications guided by the upconversion fluorescence resonance energy transfer (UC-FRET) theory [85,86].

#### 5.1.4. Cross-Relaxation (CR)

Cross-relaxation refers to the energy transfer process between identical ions or dissimilar ions with matched optical transitions, acting as both sensitizers and activators. Cross-relaxation may give rise to the following: (i) the diffusion process between sensitizers when the energy levels involved are identical (energy migration), in which no energy is lost; and (ii) self-quenching when they are different with a change or loss in the energy of the emitted photons. Since the cross-relaxation process depends on the interaction between two ions, the concentration quenching of luminescence will occur when the concentration of the activator is above a critical value.

#### 5.1.5. Photon Avalanche (PA)

The photon avalanche (PA) phenomenon was originally proposed by Chivian et al. in 1979 when studying the upconversion properties of Pr^3+^ in LaCl₃ crystals [87]. In this process, electrons are first excited to an intermediate state through a weakly non-resonant ground state and then further jump to higher energy levels using resonant excited-state absorption ESA. Between the ions in the higher excited state and the neighboring ground-state ions, two intermediate-state ions are produced by CR. By cyclically executing this cross-relaxation and excited-state absorption mechanism, the electron Brillouin number of the intermediate and excited states increases significantly. When the depletion rate of the excited state is lower than that of the ground-state ions, the so-called photon avalanche upconversion luminescence phenomenon is triggered.

#### 5.1.6. Energy Transport-Mediated Upconversion (EMU)

An interesting pathway in the upconversion field is the energy migration-mediated upconversion (EMU) mechanism, a concept pioneered by Liu et al. [88]. The key to this mechanism lies in the synergistic interactions among four rare-earth ions, a sensitizer, accumulator, migrant, and activator, each of which plays an indispensable role in the process. The process can be decomposed into several key stages: first, the energy is efficiently filled into the high excited state of the accumulator through the ETU process; then, the energy accumulated in the high excited state of the accumulator is transferred to the migrator by means of the core–shell interface of the migrator; and then, the neighboring activator ions capture the migrated energy. When the electrons receiving the energy jump back to the ground state, we can observe the radiation emission phenomenon jointly generated by the activator and the accumulator.

### 5.2. Emission Color Modulation

Rare-earth-doped upconversion nanomaterials are rich in optical and physicochemical properties. Thanks to the unique 4f electronic configuration, the emission spectra of rare-earth ions are particularly rich. Core–shell nanostructure engineering enables the fine tuning of the upconversion luminescence. By the rational design of the core–shell structure and reasonable choice of doping, researchers have been able to precisely tune the emission profiles of these nanoparticles at a variety of excitation wavelengths to produce spectra ranging from the ultraviolet and visible to the near infrared.

#### 5.2.1. Adjustment of Optically Active ion Distribution in the Core–Shell Structure

The development of core–shell structures provides the flexibility to achieve multicolor emission from core–shell nanocrystals by tuning the distribution of rare-earth optically active ions in the core–shell nanocrystals. This approach involves fine tuning the position and concentration of rare-earth elements in the nanostructures to take advantage of their unique energy level structure, leading to variations in energy transfer efficiency and the diversification of luminescent colors.

##### Homogeneous Core–Shell Structure

In recent years, homogeneous core–shell structures have demonstrated their remarkable ability to modulate luminescent colors. In their 2021 study, Zhuang et al. designed a single-doped core–shell structure (NaYF_4_: Nd^3+^/Er^3+^/Ho^3+^/Tb^3+^@NaYF_4_), which was excited by X-rays, and cleverly utilized the nuclear lanthanide doping ratio to achieve multicolor modulation of long afterglow luminescence [89]. In the same year, Zhang’s team successfully realized multispectral near-infrared long afterglow signal emission on a single nanoparticle by utilizing the unique advantage of the core–shell structure [90]. This series of results deepens our understanding of the design principles of luminescent nanomaterials and advances their potential applications in fields such as bioimaging.

##### Heterogeneous Core–Shell Structure

**Figure 6:**
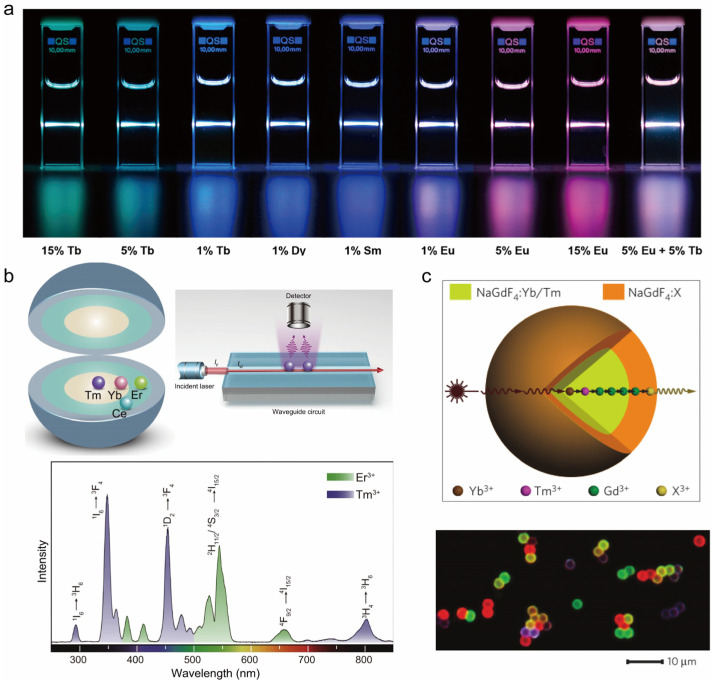
(**a**) Photograph of typical samples doped with different lanthanide ions in cyclohexane solution under 980 nm excitation. Reproduced with permission from [85]. Copyright 2012, American Chemical Society. (**b**) Schematic of NaYF_4_: Yb/Tm@NaErF_4_: Ce@NaYF_4_ structure and waveguide circuit and emission spectrum of NaYF_4_: Yb/Tm@NaErF_4_: Ce@NaYF_4_ nanoparticles under 1532 nm excitation. Reproduced with permission from [94]. Copyright 2022, Nature Publishing Group. (**c**) Schematic of NaGdF_4_@NaGdF_4_ core–shell nanoparticle structure and luminescence micrographs of polystyrene microspheres labeled with the designed core–shell nanostructures. Blue: NaGdF_4_: Yb/Tm@NaGdF_4_; Red: NaGdF_4_: Yb/Tm@NaGdF_4_: Eu; Green: NaGdF_4_: Yb/Tm@NaGdF_4_: Tb; Yellow: a binary mixture of NaGdF_4_: Yb/Tm@NaGdF_4_: Tb and NaGdF_4_: Yb/Tm@NaGdF_4_: Eu. Reproduced with permission from [88]. Copyright 2011, Nature Publishing Group.

Researchers have found that the design of heterogeneous core–shell structures has a favorable impact on the manipulation of energy flow. Su et al. delved into the effect of surface passivation on energy migration and upconversion luminescence. They found that the dissipation of excitation energy was significantly suppressed by epitaxially growing an optically inert NaYF_4_ layer on NaGdF_4_: Ln^3+^@NaGdF_4_: Ln^3+^ core–shell nanoparticles (Figure 6a) [85]. Notably, the introduction of the NaYF_4_ shell layer enables the efficient upconversion emission of a variety of luminescent lanthanide ions (Dy^3+^, Sm^3+^, Tb^3+^, and Eu^3+^) with a quite low doping concentration (1%), resulting in a wide range of luminescent colors. Zhou’s team designed the NaErF_4_: Ho (0.5 mol%) @NaYbF_4_@NaYF_4_ structure. In this structure, NaYbF_4_ was used as a sensitized layer, which significantly improved the efficiency of excitation energy capture and utilization. Trace addition of Ho^3+^ promoted the red-light kinetic process of Er^3+^. The energy transfer at the interface of Er and Yb sublattices changed the rate of the luminescence energy level layout of Er^3+^. Therefore, the dynamic adjustment of the luminescence color from red to green can be achieved by increasing the excitation power density or decreasing the excitation pulse width [91]. Zhang et al. reported a multilayered core–shell nanostructure, NaGdF_4_: Yb/Tm/Er@NaGdF_4_: Eu@NaYF_4_, which emits emission covering the entire visible range and can be combined to produce white light by optimizing the photon leap paths of specific lanthanide ions [92]. Yang and his research team proposed an innovative rare-earth sulfide/fluoride heterostructure design, which achieves efficient multicolor upconversion and downshift luminescence of rare-earth ions at a single excitation wavelength by skillfully doping different kinds of rare-earth ions [93].

UV technology has emerged in many fields, showing its wide range of applications. In ytterbium- and neodymium-doped environments, UV upconversion luminescence has been achieved through direct co-doping or structure-optimized design. However, UV luminescence has been challenging under erbium ion sensitized conditions. To address this issue, Wang et al. carefully constructed rare-earth-doped nanoparticles with a core–shell-shell structure and successfully realized UV luminescence under 1532 nm excitation (Figure 6b) through a well-designed energy transfer pathway from Er to Yb and then to Tm [94]. Their study reveals that in order to reach the cascade upconversion effect, it is necessary to rely on the design of the core–shell structure so as to avoid unfavorable interactions between active ions.

##### Active Core–Active Shell Structure

In earlier years, effective upconversion emission could only observed in Tm, Er, and Ho ions. In 2011, Wang et al. developed a Gd-based core–shell structure by core–shell structure design, which enabled the efficient upconversion emission of various lanthanide luminescent ions (i.e., Tb^3+^, Eu^3+^, Dy^3+^, Sm^3+^, and Ce^3+^) through the energy migration upconversion (EMU) mechanism (Figure 6c) [88]. Zhou and his team have proposed an innovative strategy aimed at enabling energy migration observations at the microscopic scale. The study was carried out by carefully designing a nanostructure consisting of a sensitized layer, a migrating layer, and a monitoring layer, utilizing the interfacial energy transfer (IET) mechanism to select specific sensitized ions and monitoring ions at the same time. With the help of this strategy, the team successfully observed the energy migration behavior of rare-earth ions such as Tb, Gd, and Yb at the nanoscale. The color of the emitted light gradually changed from blue to red as the concentration of Tb^3+^ increased [95].

#### 5.2.2. Adjustment of Shell Thickness

Adjusting the shell thickness of rare-earth core–shell nanocrystals is an effective strategy to realize their multicolor emission. Precise control of the shell thickness not only regulates the energy transfer path and optimizes the crystal field environment but also enhances the luminescence performance by improving the surface state, which together make the rare-earth core–shell nanocrystals able to display rich multicolor luminescence characteristics and meet the needs of multifunctional luminescent materials in different fields.

In 2023, Zhou et al. developed a core–shell nanosystem featuring a multilayer design. The core consists of NaErF_4_, with NaYbF_4_ strategically used as the sensitizer. Additionally, NaYF_4_ serves as an inert isolation layer, finely adjusting the spatial separation between the core and the sensitizer. It is found that the cross-relaxation phenomenon at the interface is effectively suppressed by this design, which affects the green light emission intensity of Er. Specifically, by adjusting the thickness of the NaYF_4_ layer, the researchers achieved precise control over the interfacial energy transfer and cross-relaxation. This precise control enabled a smooth shift in luminescence color from red to green under 980 nm excitation and maintained stable red emission under 808 nm or 1530 nm excitation, effectively managing the luminescence color [96]. Meanwhile, Wang et al. detailed the growth mechanism of core–shell nanocrystals NaErF_4_@NaYF_4_. Under the excitation of a 980 nm laser, a significant enhancement of the upconversion luminescence intensity was observed with the gradual encapsulation and growth of the NaYF_4_ shell layer. Particularly striking is that the ratio of green to red emitted light intensity changed significantly during this process [97]. The details of manipulating switchable emission colors in core–shell nanostructures are summarized in Table 2.

### 5.3. Luminescence Enhancement

When exploring the mechanism of luminescence enhancement in nanocrystals, the key points are to improve the energy transfer efficiency, reduce the non-radiative energy loss, improve the surface quality of the crystals, and precisely control the size effect. For core nanocrystals, the enhancement of luminescence can be effectively achieved by fine-tuning the surface modification, optimizing the doping concentration, and accurately controlling the size and morphology. These methods are also applicable to the luminescence enhancement of core–shell nanocrystals. In this section, we will describe the common methods for luminescence enhancement of core–shell nanocrystals, which mainly include the design of the shell layer structure, the modulation of the core–shell interface properties, and the influence of the size effect on the luminescence intensity of core–shell nanocrystals.

#### 5.3.1. Design of the Shell Structure

**Figure 7:**
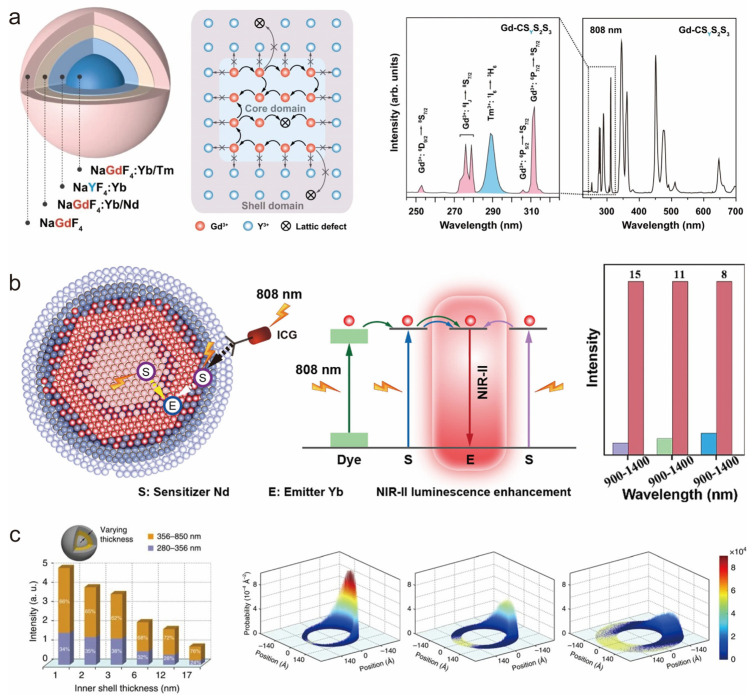
(**a**) Schematic structure of synthesized Gd-CS_Y_S_2_S_3_, schematic energy cycling of the Gd^3+^ sublattice in the core region of NaGdF_4_: Yb,Tm, and fluorescence spectra of Gd-CS_Y_S_2_S_3_ under 808 nm excitation. Reproduced with permission from [81]. Copyright 2021, Nature Publishing Group. (**b**) Schematic of the structural design and energy transfer mechanism of NaNdF_4_@NaYbF_4_@NaYF_4_:30%Nd@NaYF_4_ and the corresponding emission enhancement multiplicity of the three designed structures in the 900–1400 wavelength band. Reproduced with permission from [99]. Copyright 2023, Elsevier B.V. (**c**) Images of upconversion luminescence intensity versus inner-shell thickness and the probability distribution of the excitation energy found at the corresponding starting point as the inner-shell thickness increases. Reproduced with permission from [100]. Copyright 2016, Nature Publishing Group.

Our group systematically investigated the optical profiles of Nd^3+^-doped multilayer core–shell structures in response to excitation light at different wavelengths in 2019 [98]. We investigated the effects of multiple factors on the upconversion luminescence of Nd^3+^-doped nanoparticles, including doping concentration, core–shell structure design, excitation light source, and size effect. The upconversion luminescence efficiency under 808 nm excitation is higher than that under 980 nm excitation by adjusting the doping concentration and rationally designing the nanostructure. In the in-depth study of 2021, our team optimized the design for the core–shell structure. We proposed a new luminescence mechanism, which is based on the optical inertness of the NaYF_4_:Yb^3+^ layer to Gd^3+^, realizing the effective retention of the upconverted UV energy in the core region, successfully suppressing the energy loss due to the internal defects, and significantly enhancing the emission efficiency of the UVC (Figure 7a) [81].

#### 5.3.2. Regulation of the Properties of the Core–Shell Interface

In an in-depth exploration of energy manipulation mechanisms in core–shell nanostructures, Zhou and his research team have crafted a strategy to realize efficient upconversion and downshift emission mediated by Gd^3+^ in lanthanides through the control and modification of interfacial energy transfer. Their study further reveals that, in contrast to the energy migration process, the Gd^3+^-mediated interfacial energy transfer is actually the dominant process for photon emission in this system [53]. Recently, our group introduced a cutting-edge design for core–multishell nanoparticles that leverages co-sensitized nanostructures with the high doping of lanthanides, ion-mixing interfaces for effective energy transfer, and high absorption cross-section organic dyes for enhanced optical performance (Figure 7b) [99]. In this design, the luminescent layer (100%Yb) is strategically placed in the middle of the core–shell structure, enabling the efficient capture of excitation energy from the core (100%Nd) and the outer layer doped with 30%Nd, thus extending the energy trapping pathway and significantly boosting the down-shifting luminescence.

#### 5.3.3. Size Effect

Size effect is an important factor affecting the properties of nanomaterials. Advances in core–shell structural engineering offer the possibility of designing nanocrystals of different sizes to further enhance the optical properties of rare-earth nanocrystals. In 2016, Prof. Wang’s group reported a method to enhance the multiphoton upconversion luminescence of Yb-Tm pairs using the size effect. They designed a core–shell–shell structure with different thicknesses of luminescent layers as interlayers and found that the intensity of nanocrystalline NaYF_4_@NaYbF_4_: Tm@NaYF_4_ upconversion emission was significantly negatively correlated with the thickness of the inner layer. To delve into the intrinsic mechanism, they employed a simulated random wandering model for nanocrystals with varying luminescent layer thicknesses. The findings indicated that the likelihood of locating the excitation energy at the outset is higher when the inner layer thickness of the core–shell–shell nanocrystals is reduced (Figure 7c). This strategy of enhancing upconversion luminescence by limiting the energy migration process within the nanocrystals provides a new perspective [100]. This finding underscores the significant impact of size on energy transfer, demonstrating that by constraining the energy migration path to nanoscale dimensions, energy transfer to the luminescent center is effectively enhanced, thereby boosting upconversion luminescence. It also introduces a novel and efficient approach to leverage the size effect for improving the optical properties of nanomaterials, particularly upconversion luminescence. This enhances both the theoretical understanding and practical application of nanomaterial design and property optimization.

In a recent in-depth study, Chen et al. investigated the significant influence of size effects within nanocrystals on the energy transfer efficiency and quantum yield of upconversion luminescence [101]. They synthesized a series of three-layer rare-earth nanocrystals NaYF_4_@NaYF_4_:Yb,Tm@NaYF_4_ with different luminescent sandwich thicknesses and tested their energy transfer efficiencies and quantum yields. It was found that for rare-earth-doped core–shell nanocrystals with sizes below 50 nm, fine tuning of the thickness of the luminescent interlayer improves the energy transfer efficiency and the upconversion quantum yield is dramatically increased. Based on their results, it is demonstrated that long-range energy transfer phenomena can exist in lanthanides. This research offers insights into designing highly efficient rare-earth-doped core–shell nanocrystals. By meticulously controlling the dimensions and internal structures of the nanocrystals, particularly through optimizing the thickness of the crucial luminescent layer, the luminescence performance can be significantly enhanced. This approach not only confirms the role of size as an effective means to modulate energy transfer efficiency and quantum yield but also paves the way for broader applications of lanthanides in advanced luminescent materials.

### 5.4. Tuning Upconversion Luminescence Lifetime

Fluorescence lifetime is an intrinsic optical property of rare-earth nanocrystals, which refers to the average residence time of luminescent ions in the excited state. Rare-earth luminescent nanomaterials are a class of high-quality optical materials characterized by long lifetimes. As a high-quality luminescent probe, the lifetime of rare-earth luminescent nanomaterials can be precisely tuned by the design of various core–shell structures.

In a standard energy transfer upconversion process, the doping concentration of the sensitizer and the luminescent center plays a crucial role in determining the distance between them. The inter-ion distance greatly influences the energy interactions between them, such as cross-relaxation and energy transfer, resulting in a notable alteration in luminescence intensity or luminescence lifetime. Researchers often regulate the lifetime of luminescent ions by changing the doping concentration of sensitizers, e.g., the luminescence lifetime of Yb ions in the range of 1000 nm can be extended from 0.1 to 1.5 ms by changing the doping ratio of sensitizing ions Nd^3+^ in the rare-earth-doped nanocore–shell structure NaY_0.9_-xYb_0.1_,NdxF_4_@CaF_2_ [102]. Adjusting the doping concentration of sensitizers to regulate the lifetime of luminescent ions is an effective method for fine-tuning energy transfer efficiency and optimizing optical properties. This approach strategically controls the distance between the sensitizer and the luminescent center, thereby influencing the energy transfer kinetics. Such a strategy is crucial for designing high-performance upconversion luminescent materials. It not only enhances the luminescent properties of the materials but also offers fresh insights into the energy transfer mechanisms, paving the way for the development of innovative optoelectronic devices.

Furthermore, the luminescence decay characteristics of rare-earth ions can be affected by the epitaxial growth of inert shell layers. For instance, coating an optically inert shell layer onto the surface of a core rare-earth nanocrystal can effectively suppress its surface-related detrimental interactions, thereby resulting in an extended lifetime. The researchers found that the luminescence lifetimes of NaYF_4_:10%Yb^3+^, 30%Nd^3+^@CaF_2_ at 1000 nm were extended by two orders of magnitude (16-fold) compared with that of single-core nanocrystals under the excitation of 808 nm near-infrared light after the heterogeneous inert shell layer coating [103]. This significant extension of luminescence lifetime highlights the effectiveness of the inert shell layer coating as a means of lifetime regulation. By protecting the active luminescent ions in the nucleus from adverse surface effects, it enhances overall optical performance by optimizing the energy transfer processes. This approach offers robust strategic support for the design of high-performance luminescent materials and optoelectronic devices.

**Figure 8:**
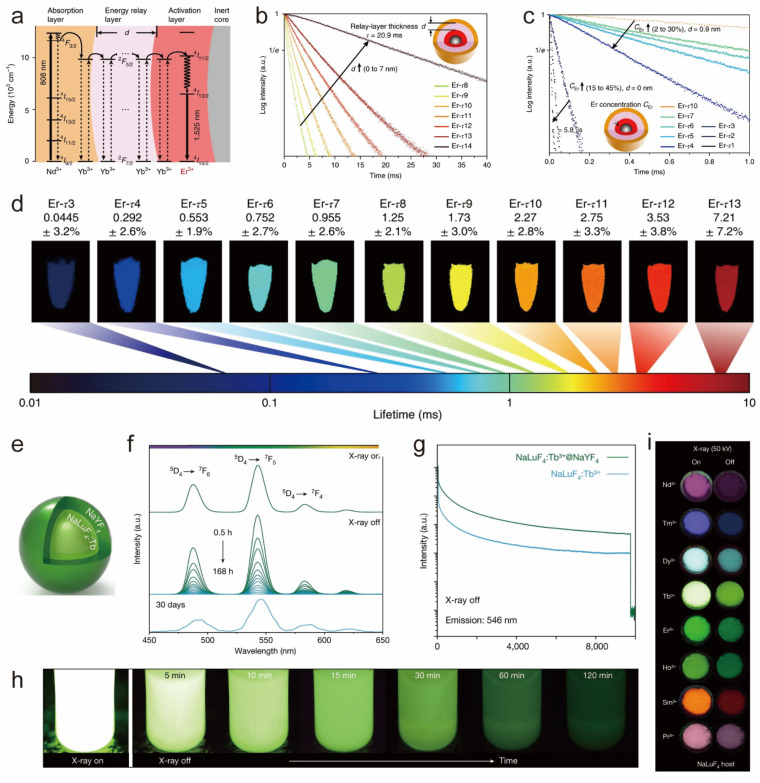
(**a**) Energy level diagrams of the luminescence processes of NaGdF_4_@NaGdF_4_: Yb, Er@NaYF_4_: Yb@NaNdF_4_: Yb. (**b**) Fluorescence decay curves of Er^3+^ with increasing shell thickness under 1532 nm excitation. (**c**) Fluorescence decay curves for Er^3+^ concentration increasing from 15% to 45% at d = 0 and from 2% to 30% at d = 0.9. (**d**) Pseudocolor-mapped lifetime images of the Er nanoparticles. Reproduced with permission from [105]. Copyright 2018, Nature Publishing Group. (**e**) Schematic structure of NaLuF_4_: Tb (15 mol%) @NaYF_4_. (**f**) Emission spectra of core–shell nanocrystals recorded for 0.5–168 h and 30 days after cessation of X-ray irradiation. (**g**) Comparison of fluorescence intensity of core–shell nanocrystals before and after coating of inert shell layer. (**h**) Afterglow photo of NaLuF_4_: Tb (15 mol%) @NaYF_4_ in cyclohexane. (**i**) Luminescence and afterglow photographs of NaLuF_4_ nanocrystals doped with different activators. Reproduced with permission from [106]. Copyright 2021, Nature Publishing Group.

In addition to cladding inert shell layers, designing active shell layers inside multilayered core–shell structures to mediate the energy transfer process can also change the lifetime of luminescent ions. For example, Zhang’s group reported a multilayered core–shell nanomaterial NaGdF_4_:25%Yb, 1%Tm@NaYF_4_:10%Yb@NaNdF_4_:10%Yb@NaYF_4_, in which the lifetime of Tm ions at 475 nm can be prolonged from 632 μs to 836 μs. Similarly, adjusting the thickness of the S1 layer in β-NaGdF_4_:20% Yb, 2%Er, 1%Pr@NaYF_4_:10% Yb@NaNdF_4_:10%Yb@NaGdF_4_ and α-NaYbF_4_:10%Er@NaYF_4_:10%Yb@NaNdF_4_:10%Yb extended Er^3+^ green and red light lifetimes, respectively [104]. In addition, they developed a series of heterogeneous core–shell structure nanocrystals NaGdF_4_@NaGdF_4_: Yb, Er@NaYF_4_: Yb@ NaNdF_4_: Yb. The rare-earth nanocrystals can adjust the lifetime of Er ions at 1525 nm in two ways (Figure 8a–d). Firstly, by increasing the thickness of the active layer (NaYF_4_: Yb), the 1525 nm fluorescence lifetime signal can be prolonged. Secondly, by enhancing the doping concentration of Er^3+^ ions in the luminescent center, the luminescent ion fluorescence lifetime can be regulated across a wide range from 5.8 µs to 20.9 ms [105]. The group’s study reveals that the luminescence lifetime of rare-earth luminescent ions can be effectively and substantially modulated through the design of active layers within a multilayer core–shell structure, by precise control over the shell layer thickness, and by varying the doping concentration of the luminescent centers. This approach offers diverse strategies for designing high-performance photonic materials with customized lifetime properties, enhancing our understanding of energy transfer mechanisms and providing a solid theoretical and experimental foundation for developing novel nanomaterials with optimized optical properties.

X-ray excitable rare-earth nanomaterials have gained increasing attention in recent years. Liu’s team in 2021 successfully designed a series of innovative rare-earth-doped nanoscintillators that exhibit excellent long afterglow luminescence properties (Figure 8e–i). They used a low-temperature co-precipitation method to finely tune the morphology and size of the rare-earth nanomaterials. By adjusting the luminescent ion species doped in the matrix, they realized spectral tuning over a wide range of spectral lines from the ultraviolet to the visible. The optical intensity of Tb-doped nanoscintillators was enhanced by 1.5 times through the coating of the inert shell layer, and the long afterglow intensity was significantly enhanced by 6.5 times [106]. The team successfully regulated the luminescence lifetime of rare-earth nanomaterials by finely tuning their size and morphology, carefully selecting luminescent ion species for spectral adjustment, and employing an inert shell layer for protection. These combined strategies led to the development of high-performance nanoscintillators with prolonged afterglow, offering innovative material solutions for X-ray excitation applications.

The engineering of core–shell structures demonstrates a powerful and flexible approach to effectively modulate the luminescence lifetime of rare-earth-doped nanomaterials, thus greatly expanding their application potential.

## 6. Conclusions and Outlook

This review systematically examines the fundamental research progress of rare-earth-doped core–shell nanocrystals, covering various key topics such as synthesis techniques, characterization methods, interfacial ion-mixing phenomena, strain effects, and the modulation of their spectroscopic properties. With the advancement in chemical synthesis technology, researchers have been able to achieve unprecedented precision in the shape control, size control, composition optimization, and surface property customization of nanocrystals, thereby successfully synthesizing the expected core–shell structures.

Exploring the atomic-scale crystal structure of rare-earth-doped core–shell nanocrystals is crucial, as it opens up a mysterious realm for rare-earth luminescent nanomaterials and represents an important part of crystallographic exploration. However, embarking on the mysterious journey to explore core–shell nanocrystal structures is undoubtedly challenging, involving not only the diversification of theoretical knowledge but also the essential support of precise characterization means, resulting in a high threshold for this exploration topic. For instance, delving into the effects of lattice strains caused by lattice constant mismatches on core–shell nanostructures is particularly challenging. These challenges stem from the requirement of atomic-level precision in synthetic and characterization techniques in terms of distribution, size, and impact area. Additionally, inducing orderly arrangement of ion-mixed interfaces and studying issues such as ion migration within the lattice also present challenges. Nonetheless, a comprehensive understanding of the nature of synthesized core–shell structures is the fundamental prerequisite for advancing theoretical knowledge and enabling more practical applications. Furthermore, there is still ample room for the exploration of rare-earth core–shell nanocrystals, and the future is filled with hope.

## Figures and Tables

**Table 1:** Synthesis and corresponding properties.

Synthesis Method	Thermal Synthesis in Water (Solvent)	Co-Precipitation Method	Thermal Injection Method
Advantages	1. Higher purity at lower temperatures. 2. Suitability for heat-sensitive compounds.	1. Simple operation, low cost. 2. No high-end equipment required.	1. Shell layer thickness can be precisely adjusted.
Disadvantages	1. Closed system hampers real-time monitoring of morphological changes.	1. More challenging in morphology control. 2. Product may contain more impurities.	1. High technical requirements: precise control of the injection volume and reaction conditions are required. 2. Equipment for precise control of temperature and injection rate is required.
Key variables and effects on characteristics	1. Temperature and pressure: dissolves insoluble materials; affects crystallization rate. 2. Solvent/ratio adjustments: control crystal growth direction, morphology, and phase. 3. Reaction time: determines phase transitions and product formation.	1. Reaction temperature: influences crystal growth rate and crystallinity, crucial for upconversion efficiency. 2. Solvent composition: enables fine tuning of epitaxial growth dynamics of the shell.	1. Injection volume: affects the thickness of the shell layer. 2. Reaction parameters: e.g., ratio of OA^−^ to OAH, amount of precursor, regulates the direction and homogeneity of the shell layer growth. 3. Temperature: affects the phase structure and degree of crystallinity of the product.

**Table 2:** Tunable emissions in core–shell nanocrystals.

Core–Shell Structural Design			Type of Activator	Emission Output	Application	Ref.
Altered reactive ion distribution	Homogeneous core–shell structure	NaYF_4_:Ln^3+^@ NaYF_4_	Tb, Er, Dy, Ho, Eu, Nd	Green, Blue, Red, Yellow, NIR	Multi-optical storage	[89]
		NaYF_4_:Nd^3+^/Er^3+^/Ho^3+^/Tm^3+^@NaYF_4_	Er, Tm, Ho, Nd	NIR	Vascular resolution, tumor imaging	[90]
	Heterogeneous core–shell structure	NaGdF_4_:Yb/Tm@NaGdF_4_:A@NaYF_4_ (A: active ion)	Tb, Dy, Sm, Eu,	Green, Blue, Red	/	[85]
		NaErF_4_:Ho(0.5 mol%)@NaYF_4_:Yb (10 mol%)@NaYF_4_	Ho, Er	Green, Yellow, Red	Optical security, speed detection	[91]
		NaGdF_4_: Yb/Tm/Er@ NaGdF_4_:Eu @NaYF_4_	Tm, Er, Eu	Green, Cyan, White, Red	Displays	[92]
		Gd_2_O_2_S:Ln^3+^@ NaYF_4_	Tb, Eu, Sm, Dy, Tm, Er, Ho	Green, Red, Pink, Cyan	/	[93]
		NaYF_4_: Yb/Tm@NaErF_4_: Ce@NaYF_4_	Tm	UV	Toroid microcavity laser	[94]
	Active core–active shell structure	NaGdF_4_: Yb/Tm@NaGdF_4_: Tb/Eu/Dy/Sm	Tb, Eu, Dy, Sm	Red, Yellow, Blue, Green	/	[88]
		NaYbF_4_:Tm/Gd@NaYF_4_:Tb@NaYF_4_:Eu	Tb, Eu	Red, Blue,	Information security	[95]
Shell thickness		NaErF_4_:Tm@ NaYF_4_@NaYbF_4_@NaYF_4_	Er, Tm	Red, Green	Information encryption	[96]
		NaErF_4_@NaYF_4_	Er	Red, Green, Orange	/	[97]

## Data Availability

All of the relevant data are available from the correspondence authors upon reasonable request. Source data are provided with this paper.
